# Differential photosynthetic and morphological adaptations to low light affect depth distribution of two submersed macrophytes in lakes

**DOI:** 10.1038/srep34028

**Published:** 2016-10-03

**Authors:** Jianfeng Chen, Te Cao, Xiaolin Zhang, Yilong Xi, Leyi Ni, Erik Jeppesen

**Affiliations:** 1 Donghu Experimental Station of Lake Ecosystems, State Key Laboratory of Freshwater Ecology and Biotechnology, Institute of Hydrobiology, The Chinese Academy of Sciences, Wuhan, 430072, China; 2 Collaborative Innovation Center of Recovery and Reconstruction of Degraded Ecosystem in Wanjiang City Belt, Anhui Province; College of Life Sciences, Anhui Normal University, Wuhu, 241000, China; 3 University of Chinese Academy of Sciences, Beijing 100049, China; 4 Department of Bioscience and Arctic Research Centre, Aarhus University, Denmark; 5 Sino-Danish Center for Education and Research, University of Chinese Academy of Sciences, Beijing 100049, China

## Abstract

To evaluate the relative importance of photosynthetic versus morphological adaptations of submersed macrophytes to low light intensity in lakes, rapid light curves (RLCs), morphological parameters, relative growth rate (RGR), clonal reproduction and abundance of two submersed macrophytes (*Potamogeton maackianus* and *Vallisneria natans*) were examined under 2.8%, 7.1%, 17.1% and 39.5% ambient light in a field and outdoor experimental study. The plants increased their initial slope of RLCs (α) and decreased their minimum saturating irradiance (E_k_) and maximum relative electron transport rate (ETRm) of RLCs under low light stress, but *V*. *natans* was more sensitive in RLCs than *P*. *maackianus*. Accordingly, the RGR, plant height and abundance of *P*. *maackianus* were higher in the high light regimes (shallow water) but lower in the low light regimes than those of *V*. *natans*. At the 2.8% ambient light, *V*. *natans* produced ramets and thus fulfilled its population expansion, in contrast to *P*. *maackianus*. The results revealed that *P*. *maackianus* as a canopy-former mainly elongated its shoot length towards the water surface to compensate for the low light conditions, however, it became limited in severe low light stress conditions. *V*. *natans* as a rosette adapted to low light stress mainly through photosynthetic adjustments and superior to severely low light than shoot elongation.

Underwater light availability is one of the most important environmental factors affecting the growth, morphology, species composition and distribution of submersed macrophytes in lakes^1^. For many submersed macrophyte species growing on different light regimes required physiological adaptations and patterns of it might be species-specific^2^. In lakes, low light stress on submersed macrophytes could be induced by many biotic and abiotic factors such as resuspension of soft surface sediment, increases in periphyton and phytoplankton, and shading of neighboring plants^3^,^4^. Eutrophication decreases the water transparency and combined with water level fluctuations, it not only affects the vertical distribution of underwater light intensity but also the amount of light reaching the sediment shifting the relative importance of nutrients versus light availability in affecting growth, distribution and species interaction of submersed macrophytes^5^,^6^. Based on the morphological characteristics of submersed macrophytes, Chambers^7^ classified them into four types of growth forms: bottom dweller, rosette, erect, and canopy former. Each form prefers different habitats relative to nutrient status and light regime, indicating species-specific adaptive strategies to the various environments. Several studies have examined the physiological and morphological responses of submersed macrophytes to various light regimes^6^,^8^,^9^,^10^. In case of low light availability in water column, submersed macrophytes might adopt one of two distinct strategies – elongation of shoot length towards water surface to alleviate low light stress and/or enhancing low light tolerance by photosynthetic adjustments. Few studies, however, have linked the two low light adaptive strategies to the distribution of submersed macrophytes along various light gradients and evaluated their relative importance.

Rapid light curve (RLC) estimates the relative electronic transport rate (rETR) as a function of photosynthetic active radiation (PAR) by using modulated chlorophyll fluorescence technology, which provides information on the temporal acclimated state of photosynthesis. The minimum saturating irradiance (E_k_), the initial slope of RLC (α) at extremely low light region, and the maximum relative electron transport rate (ETRm) inferred by the RLC reflect the ability of plants to tolerate high light, the light use efficiency and the maximum photosynthetic rate of the plants, respectively^11^. Morphological parameters, relative growth rate (RGR) and clonal reproduction provide information on the ecological adaptation of submersed macrophytes to various light regimes, because the survival, fulfillment of life history and population expansion of the plants require extra photosynthetic carbohydrate supply than those using for leaf maintenance alone^12^.

In lakes, two common submersed macrophytes, *Potamogeton maackianus* A. Benn. (canopy former)^7^ and *Vallisneria natans* (Lour.) H. Hara (rosette) grow well in waters with medium turbidity. They can both be dominant in such an environment despite their different growth forms^13^, indicating that their low light adaptive strategies differ significantly. In this study, *P*. *maackianus* and *V*. *natans* were cultured at various experimental light regimes with the aim to explore their low light adaptive strategies by measuring the RLC, morphological parameters, RGR and clonal reproduction and relating our findings to their abundance in two Chinese lakes. Specifically, we hypothesized that 1) *P*. *maackianus* and *V*. *natans* have different low light adaptive strategies as they differ in growth form, potentially leading to a trade-off between physiological and morphological responses to low light stress; 2) *P*. *maackianus* as a canopy former might alleviate the low light stress by elongating its shoot length towards the water surface, while *V*. *natans*, being a rosette with low shoot elongation capacity rather, relies on photosynthetic adjustments to cope with the low light stress; 3) photosynthetic adjustments would become more important in determining plant abundance in deep water due to the lower carbon requirement compared with shoot elongation.

## Materials and Methods

### Experiment design and layout

The experiment was conducted from 20 April to 20 July 2015 at the Donghu Experimental Station of Lake Ecosystem (30°32′53.41″N, 114°21′15.63″E) located in Wuhan City, China. In the experiment, *P*. *maackianus* and *V*. *natans* were cultured in 32 outdoor aquaria (Length: 50 cm; Width: 50 cm; Height: 80 cm) placed in a shelter covered by black nylon nets with various thickness, resulting in four light regimes – 2.8% (I1), 7.1% (I2), 17.1% (I3), and 39.5% (I4) of the air light intensities reaching the aquaria.

**Figure 1:**
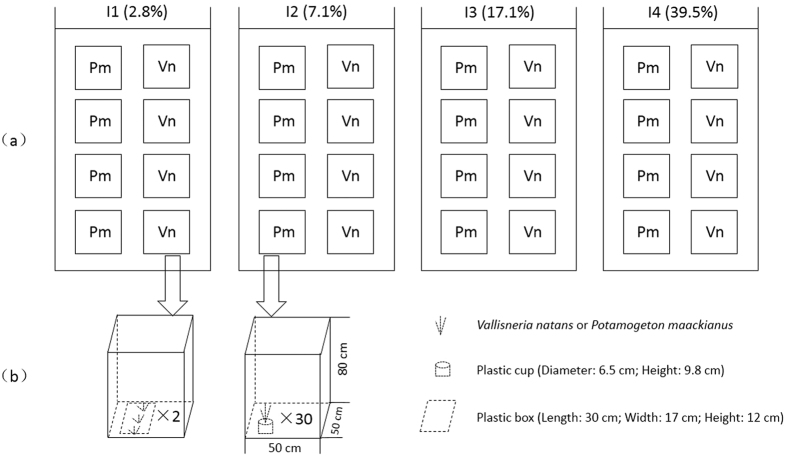
Experimental layout for the *Vallisneria natans* (Vn) and *Potamogeton maackianus* (Pm) cultivation experiment. (**a**) Aquaria (50 × 50 × 80 cm) filled with 70 cm water for cultivation of macrophytes. (**b**) The *P*. *maackianus* tanks each contained 30 cups with one *P*. *maackianus* individual in each cup, the *V*. *natans* tanks contained two boxes with three *V*. *natans* individuals in each.

At the beginning of the experiment, three healthy seedlings of *V*. *natans* (31.72 ± 8.72 cm height) were planted evenly in two plastic boxes (Length: 30 cm; Width: 17 cm; Height: 12 cm) containing 10 cm sediment collected from Lake Donghu and incubated in an aquarium filled with 70 cm water. As to *P*. *maackianus*, one healthy shoot (25.0 ± 3.1 cm height) was planted in a plastic cup (Diameter: 6.5 cm; Height: 9.8 cm) containing 9 cm sediment, and 30 cups were incubated in an aquarium filled with 70 cm water. The water used was a mixture of 70% purified water and 30% water from Lake Donghu. Four replicate treatments were carried out for each of the four light regimes (Fig. 1).

During the experiment, chlorophyll a (*Chl-a*), pH, temperature (T), and PAR was determined in the overlying water every two weeks according to the methods described by Clesceri *et al*.^14^. pH and temperature (T) were measured using a multifunctional YSI meter (Yellow Springs Instruments, Ohio, US) and PAR was measured by a Li-COR UWQ-192S sensor coupled with a Li-1400 data logger (Li-Cor, Lincoln, NE, USA) at 10:00–12:00 a.m. The mean and range (in the parentheses) were 0.48 (0.14–1.46) mg L^−1^ for total nitrogen (TN), 0.014 (0–0.075) mg L^−1^ for NH_4_-N, 0.21 (0–0.87) mg L^−1^ for NO_3_–N, 0.03 (0.01–0.08) mg L^−1^ for total phosphorus (TP), 0.011 (0.0006–0.069) mg L^−1^ for PO_4_-P and 2.48 (0–11.16) μg L^−1^ for *Chl-a* in the water. The mean and range (in the parentheses) of pH and T were 8.63 (7.42–10.24) and 25.7 (21.1–29.4) °C, respectively. The pH value of lake water nearby our experimental site was 9.08. The mean and range (in the parentheses) of PAR in the water surface in the I1 to I4 light regimes were 22.2 (9.13–43.72), 68.10 (25.57–136.45), 150.30 (60.15–294.77), and 348.89 (131.14–635.98) μmol m^−2 ^s^−1^, respectively. The contents of TN, NH_4_-N, NO_3_–N, TP, and PO_4_-P in the sediment pore water were measured at the beginning and at the end of the experiment following the method of Clesceri *et al*.^14^, and the mean contents were 1.68, 1.09, 0.34, 0.22, and 0.084 mg L^−1^, respectively.

### Determination of photosynthetic, morphological and growth parameters of the experimental plants

Ramet numbers of *V*. *natans* and *P*. *maackianus* were counted on the 30^th^, 60^th^, and 90^th^ day. The shoot length of *P*. *maackianus*, leaf length of *V*. *natans* and the branch numbers of *P*. *maackianus* were measured on day 60 of the experiment. At the end of the experiment, all plants were harvested, gently washed and dried with tissue paper and then weighted. The RGR of the plants was calculated by the formula: RGR = ln (M_2_/M_1_)/dt, where M_2_ and M_1_ were plant fresh weight at the end and beginning of the experiment, respectively and dt was the duration (days) of the experimental period.

The RLC of *V*. *natans* and *P*. *maackianus* leaves was measured by a WATER-PAM fluorometer (WALZ Company, Germany) using WinControl-3 software (v. 3.23), operating at 9 light intensity gradients (35, 79, 119, 175, 268, 396, 605, 903, and 1282 μmol m^−2 ^s^−1^), each light gradient lasting for 10 seconds. The RLC measurements were conducted at 3:00–6:00 p.m. on a sunny day (June 24) for 2 to 5 mature plant leaves in each aquarium. *V*. *natans* leaves were measured at 5 cm position from the leaf apex and the *P*. *maackianus* leaves were measured in center of leaf concave. RLC parameters were calculated by a fitting formula of White and Critchley^15^.

### Field investigation of plants abundance in response to underwater PAR

Field investigations of the abundance of *P*. *maackianus* and *V*. *natans* were carried out in Xukou Bay (ca. 8.4 km^2^; max. water depth: 2 m) of Lake Taihu (2445 km^2^) in summer 2014 and in whole Lake Erhai (250 km^2^; max. water depth: 21.0 m) in summer 2015 where these two species are the dominant submersed macrophytes^13^. Lakes Taihu, Erhai, and Donghu (the experimental site) are located in the eastern, middle and south-western parts of China, respectively and thus belong to subtropical climate zone, and all three lakes have undergone eutrophication contributing to the decline of submersed vegetation during the past three decades^16^. The concentrations of TP and TN and Secchi transparency in the water column were 0.024 mg L^−1^, 0.32 mg L^−1^, and 112 cm in Xukou Bay of Lake Taihu and 0.03 mg L^−1^, 0.75 mg L^−1^, and 153 cm in Lake Erhai, respectively.

In the field investigations, submersed macrophytes were collected by an underwater reaping hook (covering a bottom surface area of 0.20 m^2^) in triplicate at each site. In Lake Erhai, the sampling sites were set at intervals of 0.5-m water depths along transects starting from the shore to the maximum depth of plant occurrence. A total of 1200 samples were collected from 87 transects uniformly distributed around the lake shore. At transects with a steep lake bottom, submersed macrophytes were sampled at fewer sites. In the Xukou Bay of Lake Taihu, the sampling sites were set at intervals of 20-m distance along transects starting from the shore to the deepest depths where the plants occurred. A total of 108 samples were collected from 5 transects uniformly distributed around the lakeshore. *P*. *maackianus* and *V*. *natans* were separated from the collected macrophytes, gently washed, and weighed to determine the fresh biomass (FW) at each sampling site.

We measured the PAR in each sampling site at several water depth (0, 0.5, 1, 1.5, 2, 2.5 … m) for five repetition according to the actual sampling site water depth. The light extinction coefficient (K) of the water column was calculated based on the equation: K = (lnI_1_–lnI_2_)/(d_2_–d_1_), where d stands for water depth and subscript stands for water depth order: 1 is the lower position and 2 is the deeper position. I_1_ and I_2_ is PAR at water depth d_1_ and d_2_, respectively^17^. Then the K in the sediment (Ks) was obtained by fitting K in each water depth (0, 0.5, 1, 1.5, 2, 2.5 … m). Therefore, the light reaching the sediment in the field can be calculated according to the PAR in water surface (0 m), Ks and water depth. Finally, we calculated the light transmittance (PAR in sediment/PAR in water surface) and classified them into four groups of RI1, RI2, RI3 and RI4, representing groups with light transmittance values nearest to the experimental I1, I2, I3 and I4, respectively. We excluded the values bang in the middle of two treatments. The mean and range (in the parentheses) of actual water depth ranges of two species in the RI1 to RI4 in Lake Erhai were 4.78 (4.20–5.50), 2.97 (2.70–3.30), 2.14 (1.60–2.60), 0.72 (0.30–1.00) m, respectively. The mean and range (in the parentheses) of actual water depth ranges of two species in the RI1 to RI4 in Xukou Bay of Lake Taihu were 1.84 (1.80–1.87), 1.77 (1.35–1.95), 1.48 (0.90–1.85), 1.36 (1.10–1.60) m, respectively.

### Statistical analysis

Statistical analysis was carried out using IBM SPSS Statistics 19.0. One-way analysis of variance (ANOVA) was conducted to determine the statistical significance for each variable between the treatments. ANOVA results were considered significant at P < 0.05 and multiple mean comparisons were performed by Duncan’s test (at 0.05 significance level) to identify differences between the treatments. Before performing one-way ANOVA, all data were tested for normality and homogeneity. Non-normal data were Sqrt-transformed to obtain normality. Pearson’s correlations analysis was used to test for relationships between RGR and fluorescence parameters.

## Results

### The photosynthetic RLC response of *V*. *natans* and *P*. *maackianus* to the various light regimes

**Figure 2:**
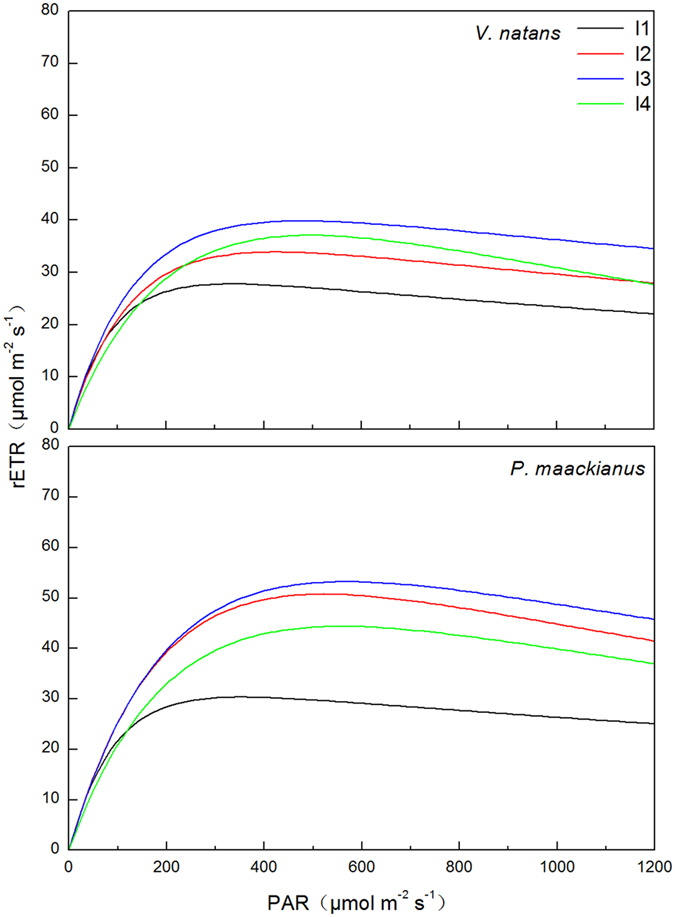
Effects of the treatments on rapid light curves (according to the formula rETR = Pm *(1 −EXP(−α* PAR/Pm)) *EXP(−β* PAR/Pm), where α, β, rETR, and Pm are the initial slope of the curve, photo-inhibition parameters, the relative electron transport rate, and the maximum potential relative electron transfer rate without photo-inhibition, respectively) of *V*. *natans* and *P*. *maackianus* on day 65 of the experiment. The I1, I2, I3 and I4 stand for 2.8%, 7.1%, 17.1% and 39.5% ambient light, respectively.

**Figure 3:**
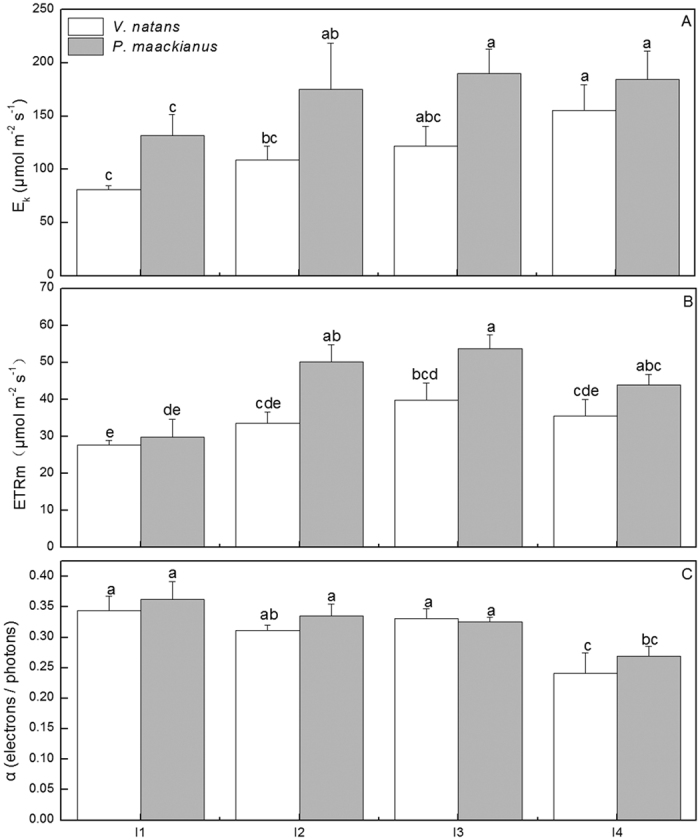
Effects of the treatments on fluorescence parameters (E_k_, α, and ETRm) of rapid light curves for *V*. *natans* and *P*. *maackianus* on day 65 of the experiment (mean ± SE, n = 4). The I1, I2, I3 and I4 stand for 2.8%, 7.1%, 17.1% and 39.5% ambient light, respectively. The A, B and C indicate parameters E_k_, ETRm and α respectively. Different letters indicate Duncan’s test at the 0.05 significance level.

The photosynthetic RLCs and their parameters (α, E_k_ and ETRm) of *V*. *natans* and *P*. *maackianus* were significantly affected by the experimental light regimes (Figs 2 and 3, F = 5.42, 6.65 and 8.76 for α, E_k_ and ETRm, respectively, and p < 0.05 for all). According to the RLCs, the rETR reached its maximum value at 333, 424, 477, and 494 μmol m^−2^ s^−1^ light intensity for *V*. *natans* (I1 to I4 light regimes) and 353, 521, 568, and 557 μmol m^−2^ s^−1^ for *P*. *maackianus* (Fig. 2). The E_k_ values of *V*. *natans* decreased gradually with decreasing light regime, with average E_k_ values of 155.4, 122.1, 109.1, and 81.3 μmol m^−2 ^s^−1^ in the I4 to I1 light regimes, respectively. The E_k_ values of *P*. *maackianus* decreased marginally when light availability decreased from the I4 to I2 regimes, and then dropped significantly when the light availability decreased from the I2 to I1 regimes, with average E_k_ values of 184.5, 189.8, 175.0, and 131.9 μmol m^−2 ^s^−1^ at the I4 to I1 regimes, respectively. The E_k_ values of *P*. *maackianus* were higher than those of *V*. *natans* in each light regime (Fig. 3A). The ETRm values of *V*. *natans* and *P*. *maackianus* showed a unimodal response to the light gradients and reached the highest values in the I3 light regime. The average ETRm values of *P*. *maackianus* were 29.9, 50.2, 53.7, and 44.1 μmol m^−2 ^s^−1^ in the I1 to I4 light regimes, respectively, and higher than the 27.8, 33.7, 39.9, and 35.6 μmol m^−2 ^s^−1^ of *V*. *natans*, particularly for the I2 and I3 light regimes (Fig. 3B). Both species had increased α when the light availability decreased, with the highest value occurring in the I1 treatment; however, the average α value in the I1, I2, and I3 treatments of *V*. *natans* increased by 36.5% as compared to I4 treatment and the average α value in the I1, I2, and I3 treatments of *P*. *maackianus* increased by only 26.3% as compared to I4 treatment (Fig. 3C).

### Morphological and growth responses of *V*. *natans* and *P*. *maackianus* to the various light regimes

**Figure 4:**
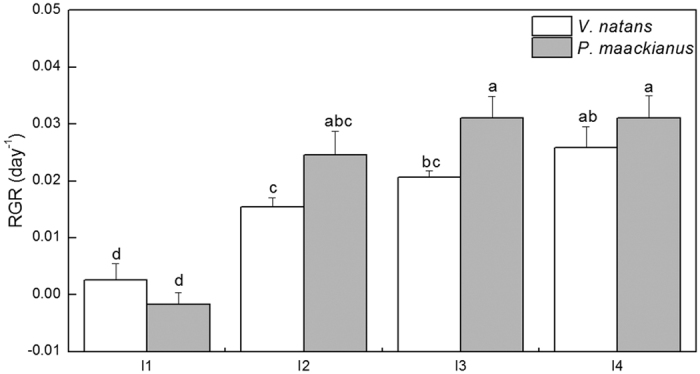
Effects of the treatments on the relative growth rate (RGR, mean ± SE, n = 4) of *V*. *natans* and *P*. *maackianus* on day 90 of the experiment. The I1, I2, I3 and I4 stand for 2.8%, 7.1%, 17.1% and 39.5% ambient light, respectively. Different letters indicate Duncan’s test at the 0.05 significance level.

The decreasing light regimes influenced the growth of *V*. *natans* and *P*. *maackianus* to a different extent. The RGR of two species were significantly affected by the experimental light regimes (F = 15.78, p < 0.05). The RGR of *V*. *natans* decreased by 90.2%, 40.3%, and 20.2% in the I1 to I3 light regimes, respectively, compared with the I4 light regime. The RGR of *P*. *maackianus* did not change significantly in the I3 light regime, exhibited a slight 21.0% reduction in the I2 light regime and declined dramatically (RGR = −0.0018) in the I1 light regime compared with the I4 light regime. The RGR of *V*. *natans* was lower than that of *P*. *maackianus* in the I2 to I4 light regimes but higher than that of *P*. *maackianus* in the I1 light regime (Fig. 4).

**Figure 5:**
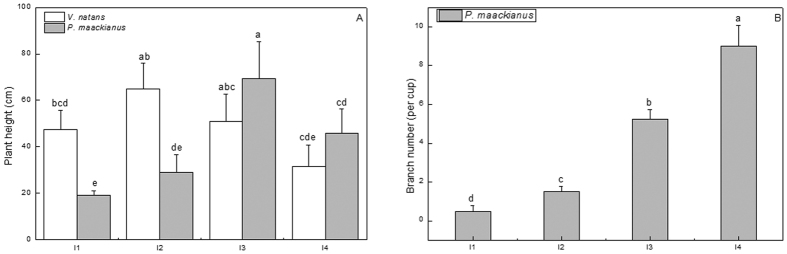
Effects of the treatments on plant height (mean ± SE, n = 4) of *V*. *natans* and *P*. *maackianus* and branch number (mean ± SE, n = 4) of *P*. *maackianus* on day 60 of the experiment. The I1, I2, I3 and I4 stand for 2.8%, 7.1%, 17.1% and 39.5% ambient light, respectively. Plant height means average maximum leaf length of each individual for *V*. *natans* and average perpendicular distance from each branch apex to stem bottom for *P*. *maackianus* in each aquarium. Branch number were the number of shoots except the highest main branch of plant in a cup. The A indicates plant height of two species and B indicates branch number of *P*. *maackianus*. Different letters indicate Duncan’s test at the 0.05 significance level.

The leaf length of *V*. *natans* and shoot length of *P*. *maackianus* were significantly affected by the experimental light regimes (F = 14.29, p < 0.05). The leaf length of *V*. *natans* and shoot length of *P*. *maackianus* showed a unimodal response to the increasing light gradients. The average leaf lengths of *V*. *natans* were highest (65.0 cm) in the I2 light regime, intermediate (mean: 49.3 cm) in the I1 and I3 light regimes and lowest (31.5 cm) in the I4 light regime. The average shoot length of *P*. *maackianus* was highest (69.5 cm) in the I3 light regime and then decreased to 45.8 cm, 28.9 cm, and 19.2 cm in the I4, I2, and I1 light regimes. The leaf length of *V*. *natans* was about twice as high as those of shoot length of *P*. *maackianus* in the I1 and I2 light regimes but 26.6% and 31.3% lower than those of *P*. *maackianus* in the I3 and I4 light regimes, respectively (Fig. 5A). The branch numbers of *P*. *maackianus* was significantly affected by the experimental light regimes (F = 26.75, p < 0.05). The branch numbers of *P*. *maackianus* increased substantially with increasing light regimes and were 3-, 10.5-, and 18-fold higher in the I2 to I4 light regimes than in the I1 light regime, respectively (Fig. 5B).

**Figure 6:**
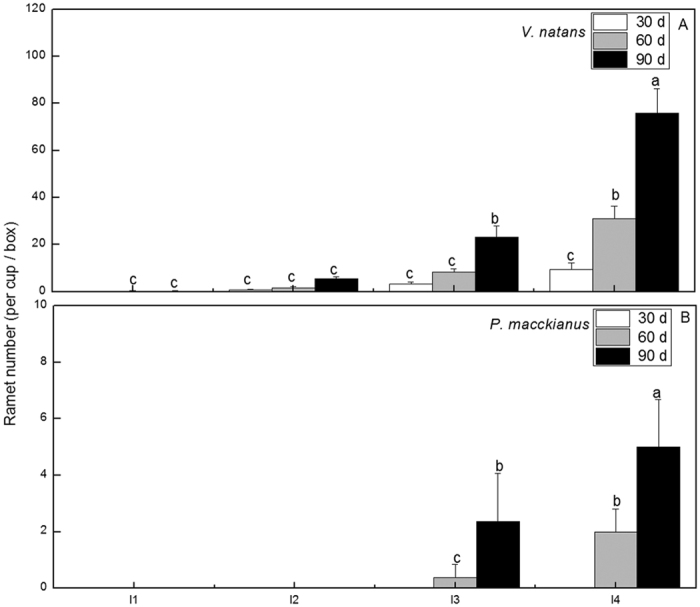
Effects of the treatments on the ramet number (mean ± SE, n = 4) of *V*. *natans* and *P*. *maackianus* on day 30, 60 and 90 of the experiment. The I1, I2, I3 and I4 stand for 2.8%, 7.1%, 17.1% and 39.5% ambient light, respectively. The A and B indicate ramet number of *V*. *natans* and *P*. *maackianus*, respectively. Different letters indicate Duncan’s test at the 0.05 significance level.

The ramet number increased substantially with increasing light intensity during the experimental period for *V*. *natans* (F = 70.53, p < 0.05) in the I1 to I4 light regimes and for *P*. *maackianus* (F = 18.02, p < 0.05) in the I3 and I4 light regimes. At the end of the experiment, *V*. *natans* had average 0.25, 5.5, 23.3, and 75.8 ramets in the I1 to I4 light regimes, respectively (Fig. 6A). *P*. *maackianus* had average 2.4 and 5 ramets in the I3 and I4 light regimes, respectively, and no ramets in the I1 and I2 light regimes (Fig. 6B).

### Abundance of *V*. *natans* and *P*. *maackianus* along light gradients in lakes

**Figure 7:**
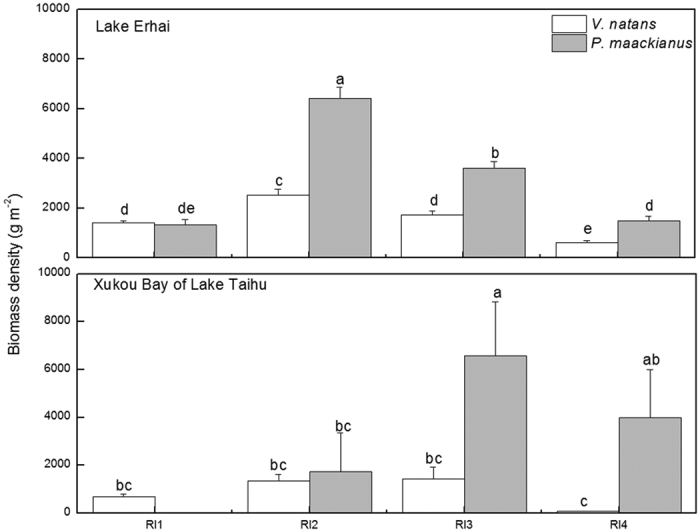
Field investigations on biomass of *V*. *natans* and *P*. *maackianus* in Xukou Bay of Lake Taihu in summer 2014 and Lake Erhai in summer 2015. The RI1, RI2, RI3 and RI4 represent light availability reaching the sediment and were equivalent to the experimental I1, I2, I3 and I4 light intensities, respectively. Different letters indicate Duncan’s test at the 0.05 significance level.

The biomass of *V*. *natans* and *P*. *maackianus* showed a unimodal response to the increasing light gradients in Lake Erhai and the Xukou Bay of Lake Taihu. The biomass of two species were significantly affected by the light regimes in Lake Erhai (F = 55.44, p < 0.05). The average biomass density of *V*. *natans* was highest (2523 g m^−2 ^FW) in the RI2 light regime, intermediate (mean: 1570 g m^−2 ^FW) in the RI1 and RI3 light regimes and lowest (629 g m^−2 ^FW) in the RI4 light regime in Lake Erhai. The average biomass density of *P*. *maackianus* was highest (6428 g m^−2 ^FW) in the RI2 light regime, intermediate (3607 g m^−2 ^FW) in the RI3 light regime and lowest (mean: 1405 g m^−2 ^FW) in the RI1 and RI4 light regimes in Lake Erhai. The average biomass density of *P*. *maackianus* was 60.7%, 51.8%, and 57.5% higher than the biomass of *V*. *natans* in the RI2 to RI4 light regimes and comparable to that of *V*. *natans* in the RI1 light regime in Lake Erhai (Fig. 7A).

The biomass of two species were significantly affected by the light regimes in Lake Taihu (F = 8.92, p < 0.05). In Xukou Bay of Lake Taihu, the average biomass density of *V*. *natans* was highest (mean: 1398 g m^−2 ^FW) in the RI2 and RI3 light regimes, intermediate (688 g m^−2 ^FW) in the RI1 light regime and lowest (75 g m^−2 ^FW) in the RI4 light regime in Xukou Bay of Lake Taihu. The average biomass density of *P*. *maackianus* was highest (6590 g m^−2 ^FW) in the RI3 light regime and then decreased to 3995 g m^−2 ^FW and 1746 g m^−2 ^FW in the RI4 and RI2 light regimes, respectively; no plants were found in the RI1 light regime. The average biomass density of *P*. *maackianus* was 4.6- and 53-fold higher than those of *V*. *natans* in the RI3 and RI4 light regimes, respectively, and comparable to that of *V*. *natans* in the RI2 light regime (Fig. 7B).

### Relationships between RGR and fluorescence parameters (α, E_k_ and ETRm)

Pearson’s correlations analysis between RGR and fluorescence parameters showed a significant positive relationship between RGR and α and E_k_ of *V*. *natans* and between RGR and α, E_k_, and ETRm of *P*. *maackianus*. For *V*. *natans*, RGR exhibited highest correlation with α. For *P*. *maackianus*, RGR exhibited highest correlation with E_k_ (Table 1).

## Discussion

The field investigations revealed that *P*. *maackianus* had higher biomass density than *V*. *natans* when light intensity was high (RI2, RI3, and RI4), while *V*. *natans* had higher biomass density in the low light stress environment (RI1). The changes in abundances of *P*. *maackianus* vs. *V*. *natans* along the underwater light gradients imply that the plants exhibit different strategies in their adaptations to the various light habitats.

The significant high positive correlation between RGR and α of *V*. *natans* suggests that the plant adjusts its physiological responses to the low light habitats (deep water) when enhancing its light capturing ability as alleviation of low light stress by leaf elongation towards the water surface become difficult for a rosette submersed macrophyte. By contrast, the significant high positive correlation between the RGR and E_k_ of *P*. *maackianus* suggests its increased photosynthetic production in the high light habitats (shallow water). The photosynthetically saturating light intensity promote shoot elongation and increased branch numbers of this canopy-forming submersed macrophyte. These results are in agreement with our first and second hypotheses.

The initial slope of RLC (α) reflects the light capturing ability of plant leaves^11^, and submersed macrophytes growing under low light stress usually have increased α value^18^,^19^,^20^. In the experiments with various light regimes, the higher proportional increases in α of *V*. *natans* indicated its better adaptation than *P*. *maackianus* to the decreased light availability. Both species tended to exhibit increased E_k_ and ETRm with increasing light intensity, which is consistent with the results of several other studies^11^,^21^, However, the lower E_k_ and ETRm of *V*. *natans* imply that relatively low light intensity may impact the photosynthetic light saturation, likely exposing the plant to excess light energy at high light intensity^18^,^20^. The higher shoot length and high branch number in the relatively high light environment induced the canopy formation of *P*. *maackianus* leading to light harvesting advantage of *P*. *maackianus* over *V*. *natans*^8^,^22^. However, the continuous spectrum of increasing E_k_ relative to the light intensities of *V*. *natans* indicated higher plasticity in its photosynthetic physiological responses to the various light regimes than that of *P*. *maackianus* whose E_k_ and ETRm were saturated at the relatively low light regimes (I2). This may contribute to the more rapid decline in *P*. *maackianus* than in *V*. *natans* abundance at the low light regimes in Lake Erhai and Xukou Bay.

In the experiments, the leaf lengths of *V*. *natans* and shoot length of *P*. *maackianus* showed a unimodal response to the decreasing light gradients, indicating that the plants adapted to the moderate but not to the extremely low light stress via leaf or shoot elongation alone. In the I1 light regime, the growth of *P*. *maackianus* was suppressed severely and its RGR became negative, meaning the plant hard to survive for prolong time, while *V*. *natans* had positive RGR and survived likely due to its photosynthetic adaptation to low light stress, reflected by the lower E_k_ of *V*. *natans* than that of *P*. *maackianus*. Similar to the leaf or shoot length responses observed in the experiments, in the field investigations the biomass of *V*. *natans* and *P*. *maackianus* exhibited a unimodal response to the decreasing light gradients, implying a close relationship between biomass and leaf or shoot length in response to various light gradients. In the lakes, increasing water depth not only supplies more space for plant growth but also induces leaf or shoot elongation due to the decreased underwater light availability, leading to linear responses in biomass to increasing water depths until leaf or shoot elongation is limited by the severe low light stress in deep water. As longer and slimmer shoots of submersed macrophyte are more prone to be damaged by hydraulic forces^23^, the photosynthetic adjustments combined with leaf elongation of *V*. *natans* may have promoted its survival under the severe low light stress conditions in Lake Erhai and Xukou Bay of Lake Taihu compared with *P*. *maackianus*.

Furthermore, the ramet production of *P*. *maackianus* was inhibited more severely than *V*. *natans* by the decreased light regimes in the experiments, thus limiting the population expansion of *P*. *maackianus* in the low light, deep water environment. It has been reported that the rosette macrophyte *V*. *natans* has a decreased photosynthetic light compensation point and an increased C reservoir in low light environments^10^,^22^,^24^ and that canopy forming macrophytes usually have a relatively higher respiration rate than rosette producers^25^,^26^. This further facilitates *V*. *natans* abundance in low light and deep water environments. These results agree with our third hypothesis.

## Additional Information

**How to cite this article**: Chen, J. *et al*. Differential photosynthetic and morphological adaptations to low light affect depth distribution of two submersed macrophytes in lakes. *Sci. Rep*. **6**, 34028; doi: 10.1038/srep34028 (2016).

## Figures and Tables

**Table 1:** Pearson’s correlation coefficient matrix of RGR and fluorescence parameters of *V*. *natans* and *P*. *maackianus* (n = 16).

Species	Parameters	α	E_k_	ETRm
*V*. *natans*	RGR	0.66**	0.59*	0.24
	α		0.79**	0.07
	E_k_			0.62*
*P. maackianus*	RGR	0.53*	0.71**	0.70**
	α		0.77**	0.43
	E_k_			0.89**

^*^Significance <0.05.

^**^Significance <0.01.
